# Supplementary material for: Patterns of Intron Gain and Loss in Fungi
Source: PLoS Biol. 2004 Nov 30;2(12):e422. doi: 10.1371/journal.pbio.0020422 (PMC532390; doi:10.1371/journal.pbio.0020422)
Supplement: Table S1 — Also available at http://genes.mit.edu/NielsenEtAl/. (4.3 MB ZIP). [file pbio.0020422.st001.zip › NielsenEtAl/html/569.html]

AN2903.1.NCU02273.1.MG00922.1.FG10782.1


```
 CLUSTAL W (1.82) Multiple Sequence Alignments - Introns Inserted


Sequence 1: AN2903.1	394 aa
Sequence 2: FG10782.1	396 aa
Sequence 3: NCU02273.1	396 aa
Sequence 4: MG00922.1	395 aa
Alignment Length: 400 aa
Number Identitical Residues: 222 aa
Alignment Score (without introns) 10367


MG00922.1 	MKGAMMTAAVLLGTAEAGVHKLKMKKIPLEDQL0KTFDLNAQMRGLGQKYLG-IRPESHQ
NCU02273.1	MKGALLTAAMLLGSAQAGVHTMKLKKVPLAEQL0ESVPIDVQVQHLGQKYTG-LRTESHT
FG10782.1 	MKSALLTAAALLGSAQAGVHKMKLNKVPLADQL0AMNSVEDHLQSLGQKYMGAARPKNAA
AN2903.1  	MKASLLTASVLLGYASAEVHKLKLNKVPLTEQF0ITRNIADHANALGQKYMGQFQQHVLE
          	**.:::**: *** *.* **.:*::*:** :*:     :  : . ***** *  : .   

MG00922.1 	QAVFSNDAVQASGNHPVPISNFMNAQY1FSEITIGTPPQNFKVILDTGSSNLWVPSSSCG
NCU02273.1	QAMFKATDAQVSGNHPVPITNFMNAQY1FSEITIGTPPQTFKVVLDTGSSNLWVPSSQCG
FG10782.1 	DYAFATNVPSVEGGHPVPVSNFMNAQY1FSEITIGTPPQSFKVVLDTGSSNLWVPSQECG
AN2903.1  	D----EPVNAMRG-HDVLVDNFMNAQY1FSEIQLGTPPQTFKVVLDTGSSNLWVPSSECG
          	:           * * * : ******* **** :*****.***:************..**

MG00922.1 	SIACYLHNKYESSSSSTYKKNGTEFKIQYGSGSMEGFVSNDVMTIGDLKIKNLDFAEATK
NCU02273.1	SIACYLHNKYESSESSTYKKNGTSFKIEYGSGSLSGFVSQDRMTIGDITINDQLFAEATS
FG10782.1 	SIACYLHSKYDSSASSTYKKNGSEFEIHYGSGSLSGFVSNDVVSIGDLKIKDQDFAEATK
AN2903.1  	SIACYLHQKFDSSASSTYKKNGSEFAIKYGSGSLSGFVSRDNLQIGDLKVKGQDFAEATS
          	*******.*::** ********:.* *.*****:.****.* : ***:.::.  *****.

MG00922.1 	EPGLAFAFGRFDGILGMGFDRLSVNKIVPPFYAMVDQKLIDEPVFAFYLAD--EKSES-E
NCU02273.1	EPGLAFAFGRFDGILGLGYDRIAVNGITPPFYKMVEQKLVDEPVFSFYLAD--QDGES-E
FG10782.1 	EPGLAFAFGRFDGILGLGYDRIAVNGMVPPFYQMVNQKLLDEPVFAFYLD--GQEGQS-E
AN2903.1  	EPGLAFAFGRFDGILGLGFDTISVNRIVPPFYNMIHQGLLDEPVFAFYLGDANKDGDSSV
          	****************:*:* ::** :.**** *:.* *:*****:*** .:.:..:*: 

MG00922.1 	VVFGGVNKDHIDGKITEIPLRRKAYWEVDLDAIALGDEVAELDNTGVILDTGTSLIALPS
NCU02273.1	VVFGGVNKDRYTGKITTIPLRRKAYWEVDFDAIGYGKDFAELEGHGVILDTGTSLIALPS
FG10782.1 	ATFGGVDKSKYTGDLEYIPLRRKAYWEVDLDAIAFGDEVAEQENTGAILDTGTSLNVLPS
AN2903.1  	ATFGGIDKDHYEGELIKIPLRRKAYWEVDLDAIALGDEVAELENTGVILDTGTSLIALPS
          	..***::*.:  *.:  ************:***. *.:.** :. *.******** .***

MG00922.1 	QLAELL~NSQIGAKKGYNGQYSIDCDKRKDLPDITFRLSGYDFPISAYDYILEVSGSCIS
NCU02273.1	QLAEML~NAQIGAKKSWNGQFTIDCGKKSSLEDVTFTLAGYNFTLGPEDYILEASGSCLS
FG10782.1 	ALAELL~NKEIGAKKGYNGQYTIECDKVSSLPDITFTLAGSNYSLPSTDYILEVQGSCIS
AN2903.1  	NLAEMI2NTEIGATKGFTGQYTIDCAKRDSLPDLTFTLTGHNFTIGPYDYTLEVQGSCIS
          	 ***:: * :***.*.:.**::*:* * ..* *:** *:* ::.: . ** **..***:*

MG00922.1 	TFMAMDIPEPVGPLAILGDAFLRRYYSIYDLGKGTVGLAKAK-
NCU02273.1	TFMGMDMPAPVGPLAILGDAFLRKYYSIYDLGADTVGIATAKR
FG10782.1 	TFQGMDFPEPVGPLVILGDAFLRRYYSVYDLGKNAVGLARAK-
AN2903.1  	AFMGMDFPEPVGPLAILGDAFLRKWYSVYDLGNGAVGLAKAK-
          	:* .**:* *****.********::**:**** .:**:* **
```
